# IQVision: An Image-Based Evaluation Tool for Quantitative Lateral Flow Immunoassay Kits

**DOI:** 10.3390/bios11070211

**Published:** 2021-06-28

**Authors:** Lalitha Pratyusha Bheemavarapu, Malay Ilesh Shah, Jayaraj Joseph, Mohanasankar Sivaprakasam

**Affiliations:** 1 Department of Electrical Engineering, Indian Institute of Technology Madras, Chennai 600036, India; jayaraj@ee.iitm.ac.in (J.J.); mohan@ee.iitm.ac.in (M.S.); 2 Healthcare Technology Innovation Centre (HTIC), Indian Institute of Technology Madras, Chennai 600036, India; malay@htic.iitm.ac.in

**Keywords:** fluorescence imaging, image quant, IQVision, medical diagnostics, point-of-care technology, quantitative lateral flow assays

## Abstract

The development of quantitative lateral flow immunoassay test strips involves a lot of research from kit manufacturers’ standpoint. Kit providers need to evaluate multiple parameters, including the location of test regions, sample flow speed, required sample volumes, reaction stability time, etc. A practical visualization tool assisting manufacturers in this process is very much required for the design of more sensitive and reliable quantitative LFIA test strips. In this paper, we present an image-based quantitative evaluation tool determining the practical functionality of fluorescence-labelled LFIA test cartridges. Image processing-based algorithms developed and presented in this paper provide a practical analysis of sample flow rates, reaction stability times of samples under test, and detect any abnormalities in test strips. Evaluation of the algorithm is done with Glycated Hemoglobin (HbA1C) and Vitamin D test cartridges. Practical sample flow progress for HbA1C test cartridges is demonstrated. The reaction stability time of HbA1C test samples is measured to be 12 min, while that of Vitamin D test samples is 24 min. Experimental evaluation of the abnormality detection algorithm is carried out, and sample flow abnormalities are detected with 100% accuracy while membrane irregularities are detected with 96% accuracy.

## 1. Introduction

Lateral flow immunoassays (LFIA) form the most widely used point-of-care (PoC) diagnostic tools. The prefabricated LFIA test strips can be employed for qualitative as well as quantitative study of the target analytes [1]. Infectious diseases like Malaria, HIV, Dengue, and non-communicable diseases, including Cardiac issues and Diabetes, etc., can be detected with these portable, one-time-use devices. In addition, LFIA technology can help in providing a quantitative estimate of haptens, proteins, vitamin and hormone levels, etc., in serum/blood samples [1,2]. Traditional diagnostic procedures like high-performance liquid chromatography (HPLC), mass spectroscopy, etc., demand expensive laboratory setups and are time-consuming. These devices also involve extensive cleaning procedures and require skilled professionals for performing the tests [3]. Contrarily, the quantitative LFIA are simple-to-use detection tools providing efficient results quickly. Moreover, LFIA test cartridges are disposed of once the testing is done and thereby prevent the risk of cross-contamination between bio-samples. Hence, they find potential applications in the field of medical diagnosis, notably in low-resource settings [2,4].

The main parameters affecting the performance of lateral flow immunoassays are good reproducibility, accuracy, and sensitivity [1,2,5]. Manufacturers need to take care of multiple factors to meet these requirements. As the LFIA test cartridges are one-time-use devices, strip-to-strip reproducibility plays a very critical role. Kit providers must evaluate and fix the LFIA test cartridge properties, including the appropriate reaction stability time, location of the test regions, sample test volumes required, etc. In addition, the capillary flow rate of the sample through the membrane forms an important parameter for test strip design. The ultimate sensitivity of an assay depends on the amount of target analyte bound with the prefabricated antigens/antibodies at the test regions. This further depends on the rate of flow of the sample, which on the other hand, impacts the reaction time as well [2,5,6]. Theoretical models that aid the manufacturers in setting the parameters mentioned above have been presented in the past [6,7,8]. These parameters, once determined, must be constant for all the test strips manufactured for a particular target analyte. However, the manufacturing tools and processes could account for a reduction in strip-to-strip reproducibility. Furthermore, improvement in the orders of the sensitivity of assays involves a lot of research and development from a manufacturing perspective [1]. Hence, there is a necessity for practical LFIA kit evaluation tools that assist the manufacturers to empirically determine the performance of fabricated test strips [1]. Especially in the R&D of fluorescence labelled quantitative test cartridges, there is a requirement for practical visualization of design parameters, including the ideal test line location, reaction stability time, quantitative measurement metrics, etc., as fluorescence quantification is not possible by directly looking at the emissions.

This paper presents an image-based visualization tool assisting the quantitative LFIA kit manufacturers towards the development of high-performance test cartridges. The developed tool is compatible with fluorescence labelled LFIA cartridges. It provides a practical analysis of sample flow properties, including capillary flow rate, practical location of the test, and control lines. The tool continuously monitors the reaction of the sample analyte at the test and control lines and updates the operator on the reaction progress. It can hence be employed to practically evaluate the optimum reaction time, the feasibility of which is discussed in [9]. There could be sample flow-related issues like the actual flow rate not being the same as per the design, or any skew in the flow arising due to obstruction, foreign particles on the membrane, flow occurring due to gravity instead of capillary action, etc. The current tool helps in practically examining these issues in the cartridges. Any irregularities present in the membrane, or the test and control lines, are also detected. This provides feedback to the operator for quality assessment of their production lot and prevents abnormal cartridges from being considered for calibration, thereby preventing faulty diagnostic measurements. The LFIA reader instruments available in the market include laser and photo-diode-based systems. However, it would be practically difficult to employ these devices for monitoring the reaction progress of the LFIA samples under test, as these involve moving mechanical components and can suffer from positioning and repeatability errors [10,11]. The hardware setup of a camera sensor-based fluorescence reader developed by our group and presented in [12] is used for the current application. The current image-based acquisition system employed does not include any moving components. With a fixed sensing and excitation mechanism, this system aids in studying the reaction progress of the sample under test, right from the time the sample is dispensed onto the test cartridge. The Cytiva Biacore systems currently available in the market help in a label-free study of biomolecule interactions, operating on the principle of Surface Plasmon Resonance (SPR). These systems mainly provide an analysis of reaction kinetics, including the association and dissociation rates of antibodies, facilitating the selection of test-specific antibodies [13,14,15]. The proposed image-based evaluation tool assists in practically determining various test membrane properties as discussed and is compatible with fluorescence-labelled quantitative lateral flow assays. Image processing-based algorithms were developed to track the sample’s reaction progress, assessing the practical sample flow through the test membrane and detecting membrane-related abnormalities. The designed tool aids the manufacturers in the development phase of quantitative LFIA test cartridges, practically examining the performance of manufactured test strips. The hardware setup, as well as the algorithms designed, are explained in further sections.

**Figure 1:**
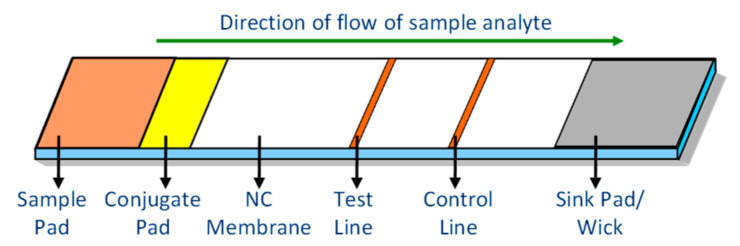
Typical configuration of a lateral flow immunoassay test strip.

The architecture of a typical LFIA test strip is indicated in Figure 1. The sample under test is dispensed onto the sample pad of the cartridge. It then migrates onto the conjugate pad, wherein particulate conjugates are immobilized, which can be fluorescent or colloidal gold or paramagnetic monodispersed latex particles [16,17]. The sample analyte remobilizes the dried conjugate, and then both migrate through the nitrocellulose (NC) membrane. The sample flows through the membrane due to the capillary action of the strip material [2]. The sample reacts with test-specific antigens/antibodies immobilized at test and control lines within the NC membrane. The sink pad/wick provides a dry region to maintain the capillary flow of the sample through the membrane as long as the liquid is present in the sample pad and prevents backflow of the sample [16]. Fluorescence-conjugated particles are advantageous over other optical labels with respect to the improved orders of sensitivity and dynamic range [18]. With fluorescence labels, it is feasible to provide a quantitative estimate of target analytes [10,19]. For this, the sample reaction, which is a function of emitted light intensity at the test and control lines, has to be measured and analyzed, which demands a reliable reader instrument. Quantitative measurements are made once the sample reaction becomes stable.

## 2. Materials and Methods

### 2.1. IQVISION Hardware Architecture

**Figure 2:**
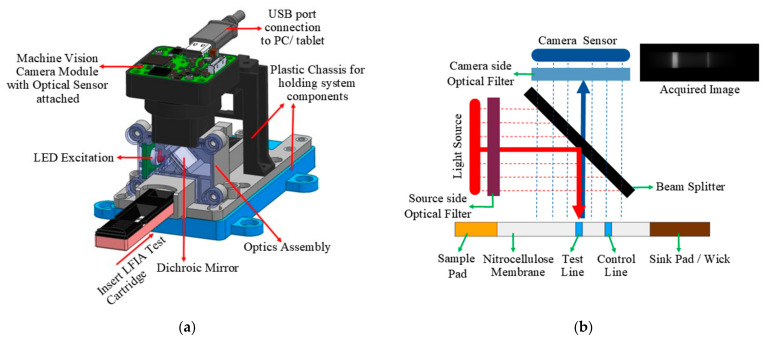
(**a**) Hardware setup of the fluorescence reader indicating different components of the system and the confocal arrangement. (**b**) Principle of operation of the designed system.

The hardware architecture of the designed LFIA reader is as indicated in Figure 2. The main components of the system design include the excitation source, the confocal optical arrangement, and the sensing module. A high-power red LED source R42180-06 (Seoul Semiconductor, Ansan-si, Korea) with a 127° view angle emitting 630 nm wavelengths is used for excitation. To filter the narrowband excitation from LED, a passband filter is used on the source side (Omega Optical Inc., Brattleboro, VT, USA), passing wavelengths below 640 nm. The LFIA cartridge under test is placed at the bottom side of the optical reader, as shown in Figure 2a. The camera captures corresponding fluorescence emitted from the cartridge, which has a wavelength of around 665 nm. For facilitating this, the excitation source and the sensing modules are fixed in a confocal arrangement, as shown in Figure 2b. It primarily involves a dichroic mirror (Omega Optical Inc., USA), which reflects wavelengths below 650 nm and passes wavelengths greater than 650 nm. It is placed at a 45° angle between the source and sensing element. The dichroic mirror reflects the excitation beam incident at 45° to its surface onto the cartridge. Similarly, the emitted fluorescence incident at a 135° angle to the mirror can pass through it. A sharp, custom-made bandpass filter (Omega Optical Inc., USA), which passes wavelengths between 655 nm to 721 nm, is placed on the camera side. When a cartridge is inserted into the reader, the LED excitation source is turned on. The camera side filter, along with the dichroic mirror, helps in capturing only the emitted fluorescence, eliminating the background. The above-mentioned light excitation source and the filter cut-offs are selected based on the Alexa Fluor 647 dye used in our LFIA cartridges. Different manufacturers can use a different dye for fluorescence. Based on the dye chosen, the light source and filter cut-offs can be changed. The overall system arrangement remains the same.

A complementary-metal-oxide type machine-vision camera sensor (Imaging Development Systems, Obersulm, Germany) with a resolution of 6.4 MPixel was used for capturing the fluorescence. It has a dynamic range greater than 75 dB and is well suited for low-contrast applications. The camera sensor module has an on-chip 12-bit analog-to-digital converter to convert the analogous light intensities to digital images. The captured images are then transferred to a personal computer/tablet through a USB interface. An acquisition system and graphical user interface (GUI) was developed in LabVIEW (National Instruments Corporation, Austin, TX, USA) to access the camera module. The designed GUI provides the user with the controls of exposure time, gain, and frames to be captured. Materials used for the study include HbA1C and Vitamin D LFIA cartridges (J Mitra & Co. Pvt. Ltd., Delhi, India), test-specific buffer and conjugate solutions (J Mitra & Co. Pvt. Ltd., India), micropipette (Thermo Fisher Scientific, Waltham, MA, USA), and micro tips.

### 2.2. IQVISION Algorithm Development

**Figure 3:**
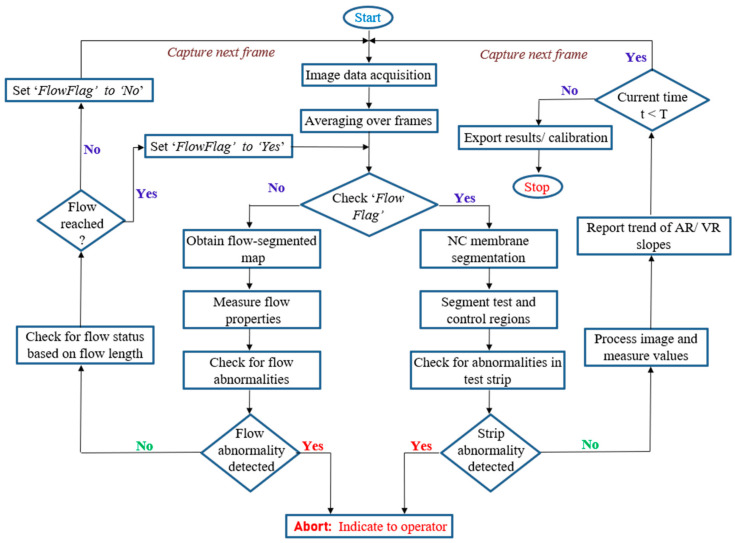
Flowchart of the designed algorithm indicating the steps involved in the algorithm execution.

The flow chart of the designed algorithm is depicted in Figure 3. This algorithm monitors the reaction progress of the sample under test right from the time it is dispensed onto the cartridge, including the tracking of sample flow through the membrane. A detailed explanation of various blocks involved in the algorithm is as follows.

#### 2.2.1. Image Data Acquisition and Noise Removal

Image data are acquired starting from time t = 0 min to a time ‘T’ minutes as specified by the user. Images are acquired for every 20 s time interval. In low-contrast applications, there is a substantial amount of shot noise present. The current setup involves imaging of static objects alone, and in such applications, we can eliminate the shot noise by averaging over multiple frames, typically greater than two [20]. Hence, at each test instance, i.e., for every 20 s time interval, images are acquired in burst mode to capture five frames of data, with a frame rate of 10 Hz. The five image frames captured are averaged to a single image I(t) and then fed to further blocks.

#### 2.2.2. Tracking of Flow Progress

Once the sample is dispensed onto the sample pad of the test cartridge, it passes through the NC membrane due to the capillary action. Within the initial few minutes of the test, sample flow through the membrane is analyzed. The flow of sample analyte through the membrane is detected through the designed algorithm and verified for any abnormal flow conditions.

Flow progress within the NC membrane is determined by segmenting out the flow region of the captured image. Here, the concept of global thresholding is adapted, wherein pixel values of the input image I(t) greater than the set threshold intensity (T) are assigned a binary ‘1’ value, and the rest are mapped to binary ‘0’ as indicated [21].

**Figure 4:**
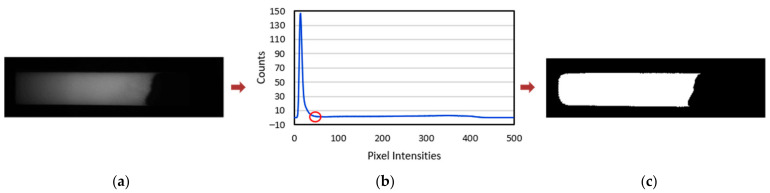
(**a**) Sample image frame captured during the flow process. (**b**) Corresponding histogram plot indicating the valley considered for thresholding. (**c**) The resulting binary map obtained from the global thresholding process.

FM(t)(x,y) = 1, if I(t)(x,y) > T = 0, elsewhere(1)

where FM(t) is the binary flow map obtained and (x,y) indicates the pixel location in the 2D image. Threshold values for segmentation are determined dynamically based on the corresponding histogram. Figure 4b depicts the histogram plot for a sample input frame. The largest valley after the first dominant peak is considered for determining the sensitivity for thresholding.

**Figure 5:**
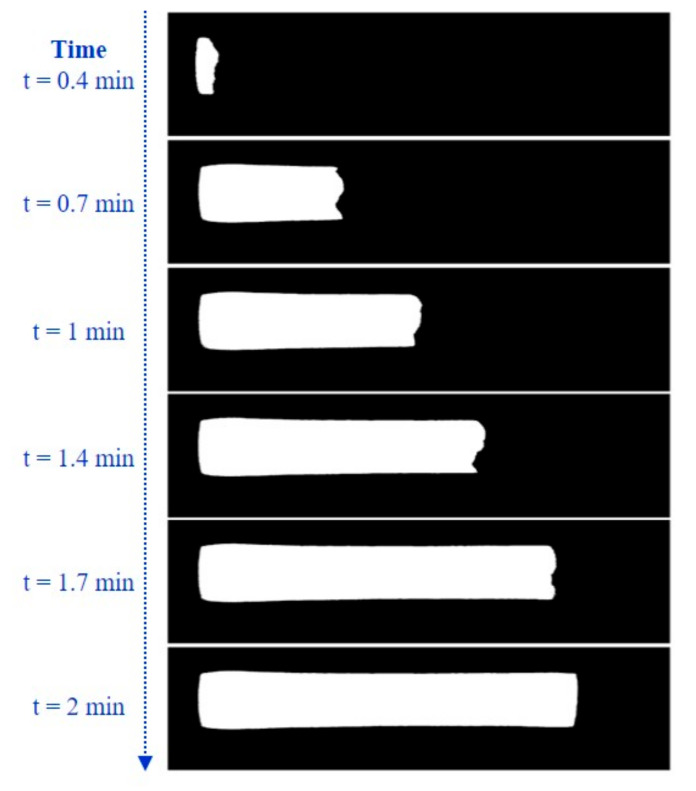
Binary maps obtained with time as the sample flow progresses through the NC membrane.

From the obtained binary map for the input image, pixels are verified for 8-neighbourhood connectivity. Pixels are grouped into individual segments if they are connected horizontally, vertically, or diagonally. Hence, all the pixels corresponding to the flow are grouped into a single segment. There could be few undesired random dot-sized pixels that are segmented as binary ‘1’. To eliminate these pixels and segment out only the flow area of interest, we apply an area filter to consider only the components with an area size greater than 0.003 mm^2^. This is evaluated for each of the averaged image frames captured for every 20 s, and the track of the sample flow through the NC membrane is observed. Figure 5 indicates the flow progress as captured for HbA1C test cartridges.

**Figure 6:**
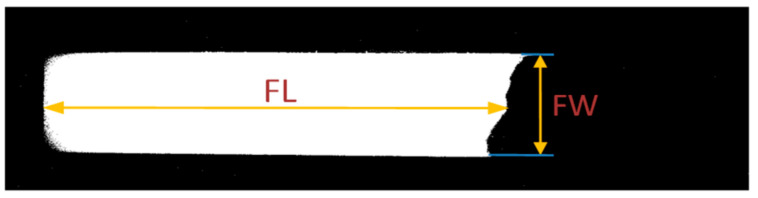
The flow properties–length and width as obtained from the binary map.

The properties, including width, length, and area of the segments, are evaluated as the sample flow progresses and verified for any irregularities. Flow length (FL) is evaluated by determining the number of pixels in the mid-row of the extracted flow segment as indicated in Figure 6. Similarly, flow width (FW) is the number of pixels in the mid column of the segment, while the flow area (FA) is the total number of pixels in the segment. Ideally, as the sample flows through the membrane, the flow length and flow area start increasing and stabilize after a few frames. Flow width should remain constant throughout and should be the same as the width of the NC membrane. The speed of the sample flow within the membrane also impacts the reaction at test and control lines. The frame-to-frame flow speed (FS) between the test instances is measured by determining the change in flow length (FL) within the time interval as indicated in (2).

(2) $$\mathrm{FS}\left(k\right)=\frac{\mathrm{FL}\left(k\right)-\mathrm{FL}\left(k-1\right)}{T\left(k\right)-T\left(k-1\right)}$$

where k indicates the current frame and T(k) is the time at the kth frame.

#### 2.2.3. NC Membrane Segmentation

**Figure 7:**
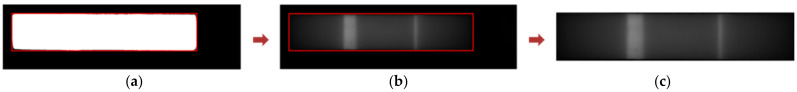
Segmentation of the NC membrane. (**a**) Obtaining the bounding box from the final flow map. (**b**) Mapping the obtained bounding box onto the input image frame. (**c**) Segmented out NC membrane.

We track the sample reaction progress at the test and control lines once the sample flow front reaches the sink pad. However, as the sink pad is not within the visible window of the cartridge, the time when the sample reaches the end of the NC membrane is taken as the reference. This is where the sink pad starts, as depicted in Figure 1. Hereon in this paper, we will be referring to this as ‘flow reached’ as a flag to start the tracking of reaction progress. For this, the NC membrane is segmented from the input image frames, within which the test and control areas are identified. Once the flow is reached, the corresponding binary flow map obtained is used for the NC membrane segmentation. The bounding box of this final flow map is determined and mapped onto the input image frames for extracting the NC membrane, as indicated in Figure 7.

#### 2.2.4. Segmentation of Test and Control Regions

For the detection of test and control areas within the segmented NC membrane, the algorithm presented in [9] is adopted. It works on the concept of binary thresholding. An experimentally determined threshold value (RT) is set, and the corresponding binary map is obtained.

(3) $$B\left(t\right)\left(x,y\right)=1,\;\mathrm{if}\;\mathrm{NCM}\left(t\right)\left(x,y\right)\;>\;\mathrm{RT}\;\;=0,\;\mathrm{else}$$

**Figure 8:**
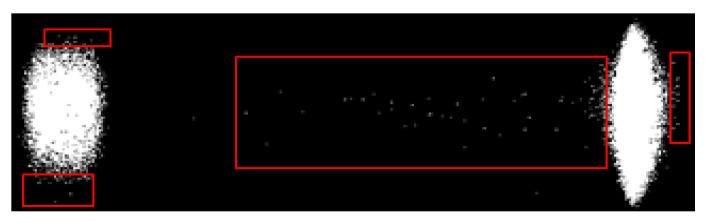
The dot-pixels obtained from the image thresholding. These are removed by applying an area filter over the acquired map.

Here, NCM (t)(x,y) and B(t)(x,y) indicate the fed NC membrane and the corresponding binary map obtained at a time ‘t’, and (x,y) indicates the pixel location and the reaction threshold value set is indicated as RT. The binary map B(t) obtained has to be further processed to appropriately extract the individual test and control area components by verifying the pixel connectivity. Each of the components is labelled and can be independently accessed. To eliminate the dot-pixels segmented as indicated in Figure 8, an area filter is applied. The resulting area-filtered binary map AFB (t)(x,y) should ideally consist of two components corresponding to the test and control regions, which are classified, respectively, by determining the center locations of the components. The test and control regions’ segmented image (SI (t)(x,y)) is derived by multiplying the binary map with the input frame I(t)(x,y) as indicated in (4)

(4) $$\mathrm{SI}\left(t\right)\left(x,y\right)=I(t)(x,y)\;*\;\mathrm{AFB}(t)(x,y)\;$$

**Figure 9:**
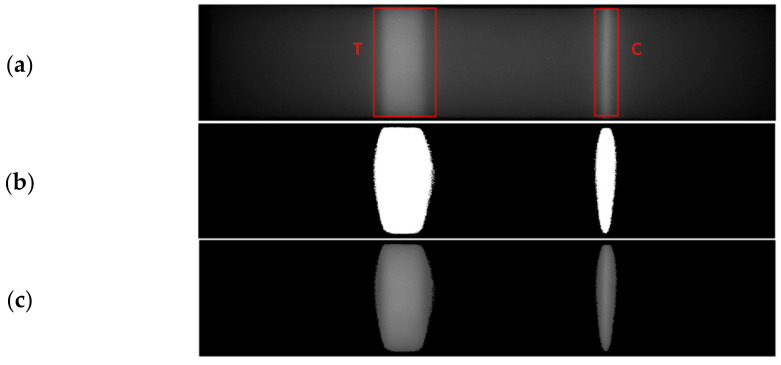
(**a**) The test (T) and control (C) lines indicated for a sample image captured. (**b**) Corresponding binary maps obtained. (**c**) Test and Control regions segmented out.

**Figure 10:**
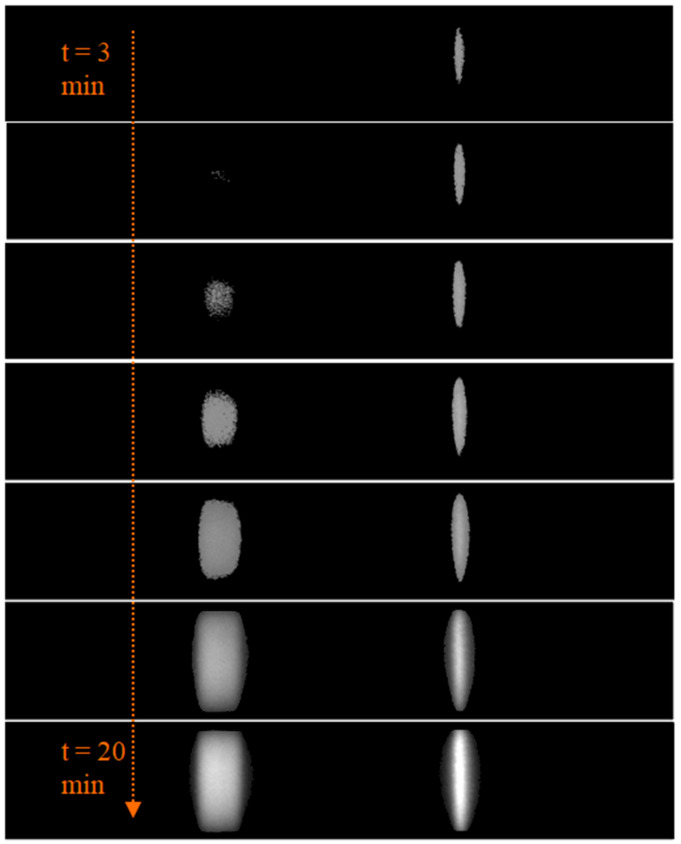
The intermediate segmented test and control regions obtained for tracking the reaction progress of sample under test once the sample flow is complete, from time t = 3 min to t = 20 min.

From the segmented image, as shown in Figure 9, the test and control region properties are measured and tracked to indicate the reaction progress of the sample under test. The total number of pixels within each of the test and control regions is determined and referred to as the test area (AT) and control area (AC), respectively. Similarly, test and control volumes VT and VC, respectively, are calculated as well by summing the pixel intensities in the test and control areas as indicated in (5) and (6).

(5) $$V_{T\;}=\overset{A_{i}}{\underset{i=1}{\sum }}\mathrm{TC}(i)$$

(6) $$V_{C}=\overset{A_{c}}{\underset{i=1}{\sum }}\mathrm{CC}(i)$$

where TC (i) is the intensity value of the individual pixels in the test region component. Similarly, CC(i) is the intensity value of the individual pixels in the control region component. Figure 10 depicts the intermediate segmented images obtained for an HbA1C test sample, indicating how the test and control regions grow with time. For a quantitative estimate of the samples, the ratio of test and control areas as well as the test and control volumes referred to as area ratio (AR) and volume ratio (VR), respectively, are evaluated as indicated.

(7) $$\mathrm{AR}=\frac{A_{T}}{A_{C}}$$

(8) $$\mathrm{VR}=\frac{V_{T}}{V_{C}}$$

**Figure 11:**
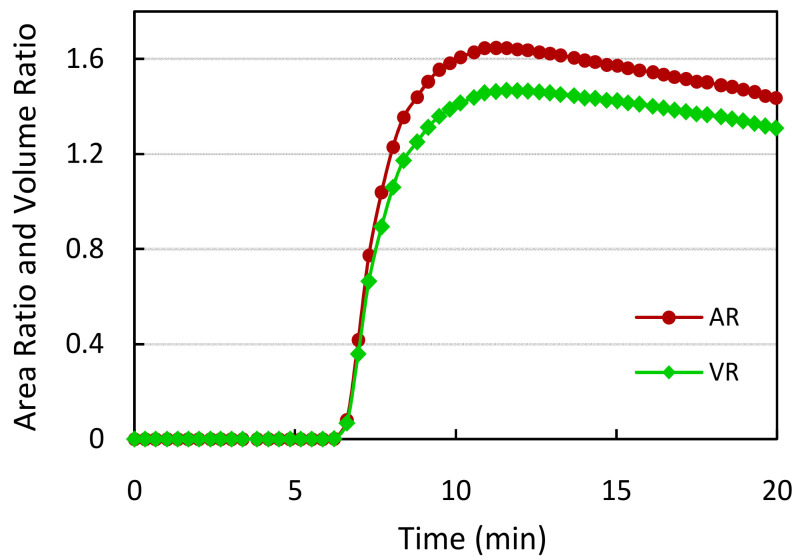
The trend of area ratio (AR) and volume ratio (VR) values measured with increasing time for a sample HbA1C test cartridge.

For each input image frame fed every 20 s, the test and control regions are segmented once the sample flow through the membrane is reached. Corresponding AR and VR values are measured for tracking the reaction progress. During the time of the sample flow, the AR and VR values are zero. Later, the AR and VR values initially increase at faster rates, as indicated in Figure 11, and after a specific amount of time, the values vary at reduced slopes. The quantitative measurements are to be made once the reaction becomes stable when the change in AR and VR values with time is almost constant. The designed algorithm aids in the development of LFIA test kits, wherein the manufacturers can experimentally evaluate the reaction stability time.

#### 2.2.5. Detection of Abnormalities

For an accurate quantitative analysis of the samples, the LFIA test strip must be free from any irregularities, which could be flow-related issues or some undesirable bright/dark regions in the test and control regions or any constrictions within the NC membrane, etc. The bright/dark regions are observed in the captured image frames due to improperly manufactured test strips or damaged membranes, which could lead to incorrect results. The algorithm was designed to detect and notify the operator of any such irregularities in the test cartridge, providing a qualitative check for cartridge manufacturing.

1. Abnormalities in Sample Flow

As mentioned earlier, sample flow through the NC membrane is due to the capillary effect, which contributes to the uniform flow of sample across the test and control lines, where the antigen–antibody binding occurs. Therefore, the capillary flow plays a critical role in determining the sensitivity. Any obstruction ceasing the flow can be detected by continuously monitoring the flow parameters—flow length, area, and width mentioned in Section 2.2.2.

**Figure 12:**
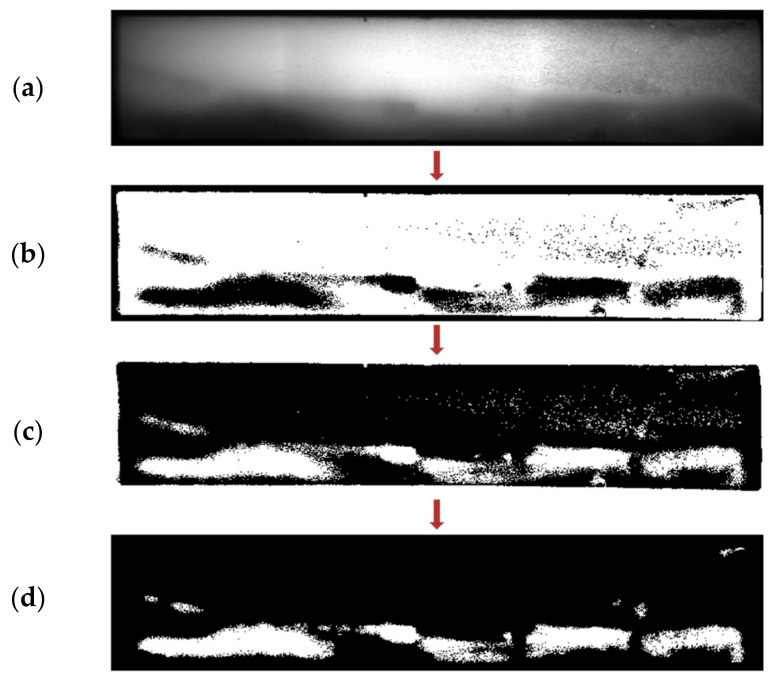
The step-by-step binary maps obtained for the detection of flow skewing with a faulty sample cartridge. (**a**) The captured image of the faulty cartridge. (**b**) Obtained binary map. (**c**) Inverted binary map making the skewed flow foreground. (**d**) Final processed flow segmented map obtained, the area of which is calculated to verify for abnormalities.

Apart from this, skewing of the sample flow through the membrane, as indicated in Figure 12, is another undesired outcome of irregularly manufactured strips. For detecting this, the concept of adaptive thresholding is adopted [22,23]. Unlike global thresholding, where a single fixed threshold is applied across the image, adaptive thresholding involves different threshold values set locally for each pixel of the image. The values for the threshold at each pixel location depend on the neighboring pixels and thus can be employed to detect randomly occurring irregularities. The threshold at each pixel of the image is computed by determining the local mean intensity within the pixel’s neighborhood over a preset window size (w × w). The local neighborhood mean (µ) in a w × w neighborhood region R, centered at the location (x,y), is calculated as

(9) $$\mu (x,y)=\frac{1}{N}\;\underset{\left(x,y\right)\;\in R}{\sum }I\left(x,y\right)$$

where I is the input image frame and N is the number of pixels in the neighborhood, N = w × w. If a pixel value is less than its corresponding local mean value by ‘t’ percentage, it is mapped to binary ‘0’, otherwise to ‘1’ as indicated in Figure 12b. The value of ‘t’ is preset. The binary map obtained is inverted, i.e., subtracted from 1, making the skewed flow foreground, as shown in Figure 12c. The dot-pixels and the corner segments are trimmed as in Figure 12d. The final flow segmented map area is calculated, and if it is greater than 5% of the map size, the irregularity is detected, and the user is alerted.

2. Irregularities in Test Cartridge

**Figure 13:**
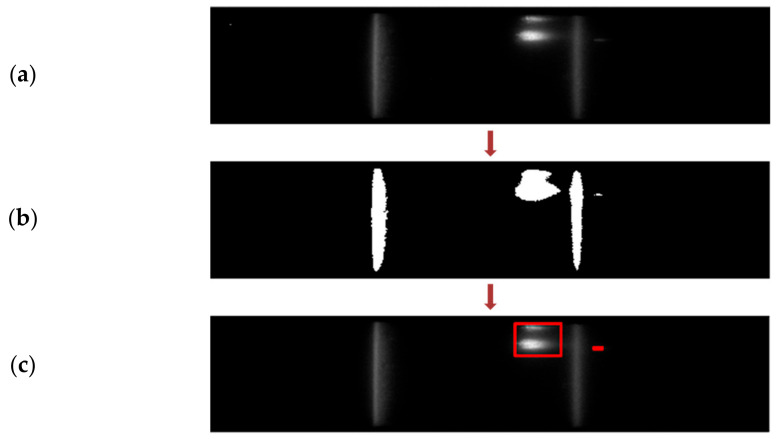
(**a**) Image frame captured for the detection of bright spots within NC membrane. (**b**) Corresponding binary map obtained. (**c**) Anomalies detected with the algorithm.

One of the typical irregularities can be any bright spots present on the NC membrane, apart from the test and control regions, as indicated in Figure 13a. The global thresholding algorithm mentioned before for segmenting the test and control lines can be used to detect these irregularities. Once the algorithm is run, it verifies the number of segmented components and their corresponding centroids. Ideally, there should be a maximum of two components pertaining to the test and control lines. However, if any additional component is identified, it indicates the presence of some bright regions. Additionally, verifying the centroid of each component distinguishes the irregularities from the test and control lines.

3. Presence of Bright/Dark Regions within Test and Control Lines

**Figure 14:**
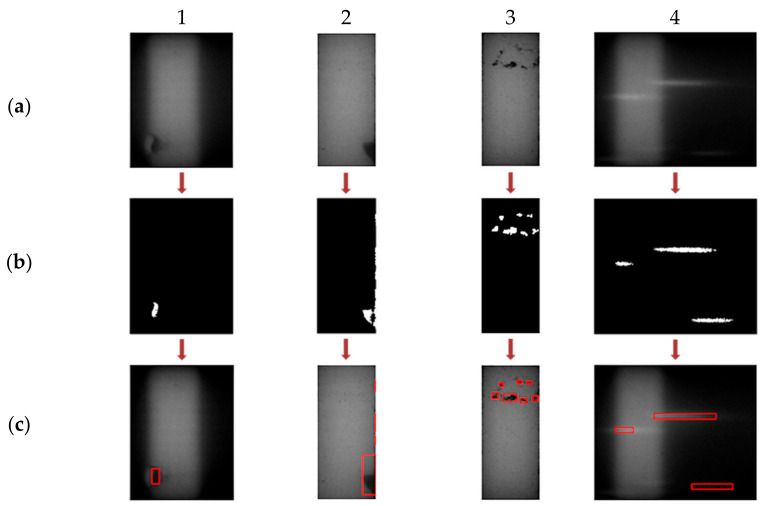
(**a**) Image frames obtained for detecting the irregularities within the test and control regions. (**b**) Final binary maps obtained. (**c**) User alerted of the detected abnormalities.

Once the test and control regions are segmented, the designed algorithm also checks for any undesired bright or dark regions present within these regions, as indicated in Figure 14. Here as well, the detection is done through adaptive thresholding mentioned earlier. The binary map indicated in Figure 14 is obtained by mapping the pixels to binary ‘0’ and ‘1’ values to identify bright regions. Similarly, the obtained binary map is inverted for detecting dark regions, making the dark pixels the foreground. The number of connected components is determined from these final binary maps, as explained in Section 2.2.2. In the ideal case, where there are irregularities within the test and control lines, the number of components would be 0. If any segmented components are identified, the user is alerted and indicated, as shown in Figure 14.

## 3. Results and Discussion

Evaluation of the designed algorithm is carried out with LFIA cartridges from our industrial partner J Mitra & Co. Pvt. Ltd., India. The test cartridge is inserted into the fluorescence-based LFIA reader described in Section 2. Images are acquired for every 20 s time interval for a time fixed by the user. At each image capture instance, five frames of image data are acquired in burst mode. This is to perform averaging over multiple frames as explained in Section 2.2.1 to eliminate shot noise. The tests are conducted in compliance with the WMA Declaration of Helsinki.

### 3.1. Evaluation of Sample Flow through the Membrane

**Figure 15:**
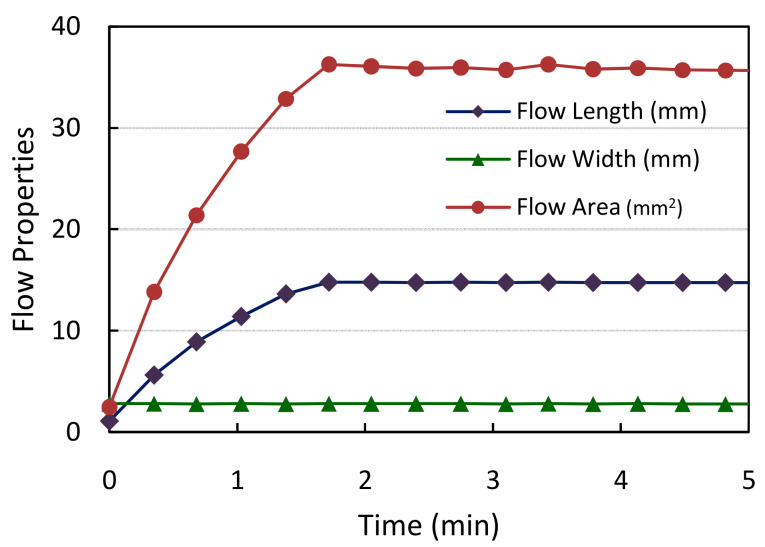
The measured flow length, width, and area for a sample HbA1C cartridge with respect to time using blood samples.

The algorithm initially monitors the flow of the sample through the NC membrane and verifies any abnormalities. Experimental evaluation was carried out for HbA1C test cartridges using whole blood samples. The trend of flow properties, including the flow length, area, and width with increasing time observed for a single test sample for the current set of cartridges employed, is indicated in Figure 15. The resolution of the images captured in terms of pixels/mm is 140. Based on this, the obtained flow properties are converted into physical dimensions (mm). It can be observed that the flow length and area values gradually increase as the flow progresses and are stabilized after a few minutes once the sample reaches the end of the membrane. The width of the sample flow remains constant throughout the span as indicated, signifying a properly manufactured NC membrane strip.

**Figure 16:**
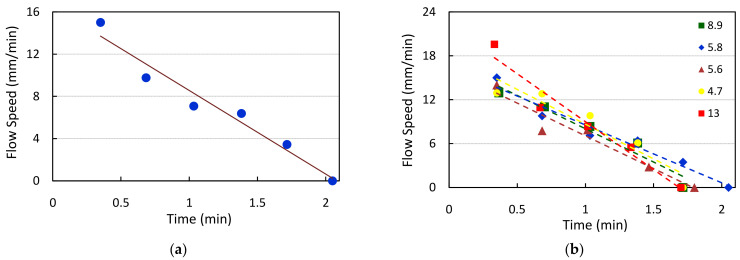
(**a**) The frame-to-frame variation in flow rate as captured for a single HbA1C test cartridge. (**b**) The trend of the frame-to-frame flow rates observed for different HbA1C test cartridges of varying concentrations.

The algorithm also provides the manufacturer with information on the flow speed of the sample—this aids in the practical evaluation of the capillary flow of the NC membrane material. Figure 16a indicates the frame-to-frame change in the flow speed for a particular cartridge under test. It can be observed that flow speed decreases linearly and becomes zero at around time t = 2 min. This provides the manufacturer with a track of how the sample flow rate is practically occurring. Any deviation from this trend, including the time taken for the flow to reach, can be observed during the test cartridge design and development phase. The flow speed measurements done for five different test samples are indicated in Figure 16b. We can notice that the sample flow is reached for all the test cartridges within a time interval of 1.5–2 min. Based on these measurements, the manufacturer can make a relevant decision on whether any modifications in the design are required.

### 3.2. Segmentation of Test and Control Lines

#### 3.2.1. Analysis of HbA1C Test Samples

Once the sample flow is reached, the algorithm performs NC membrane segmentation with the bounding box obtained from the flow measurements as a reference, as mentioned in Section 2.2.3. The reaction progress of the sample is then illustrated to the operator by segmenting out the test and control regions within the extracted NC membrane and measuring the corresponding volume ratio (VR) values. The initial evaluation was done with HbA1C test samples. For each test, a 5 µL of blood sample is mixed with test specific buffer and dispensed on the cartridge, and the algorithm is run. Images are acquired continuously for 20 min, with a capture interval of 20 s. Hence, for a single test, 60 image sets with 5 image frames each are captured, meaning a total of 300 images are processed for every run. Ten different whole blood samples were used for experimentation, with varying HbA1C concentrations ranging from 5–14%. This involves a total sample data of 3000 images.

**Figure 17:**
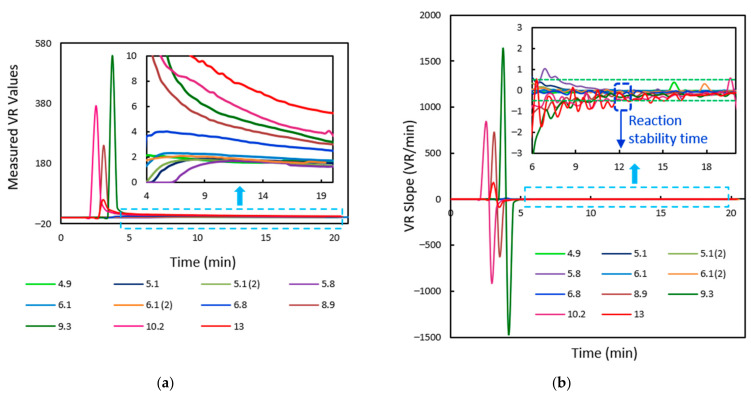
(**a**) The volume ratio values measured for 10 HbA1C test cartridges of different concentrations. The inset image depicts the graph zoomed-in from time t = 4 min to t = 20 min. (**b**) The corresponding trend of change in VR slopes with time, the zoomed-in graph from time t = 6 min to t = 20 min indicated as well.

The NC membrane and the test and control regions were observed to be properly segmented out for all the test samples through the algorithm explained in Section 2.2.4. The trend of measured volume ratios of the samples is depicted in Figure 17a. Initially, the values vary at higher rates and slowly tend to change at decreased slopes. The rate of change of VR values with respect to time is also observed, as indicated in Figure 17b. For defining the stability time of the test, we verify for the time when the rate of change of VR values with time is almost zero. Since this may not be practically feasible, the time instant when the VR slopes for 95% samples fall within ±0.5 span is considered for further examination. For the current lot of test cartridges, this is found to occur at around 12 min. This is the time where the quantitative measurements are to be made.

#### 3.2.2. Analysis of Vitamin D Test Samples

**Figure 18:**
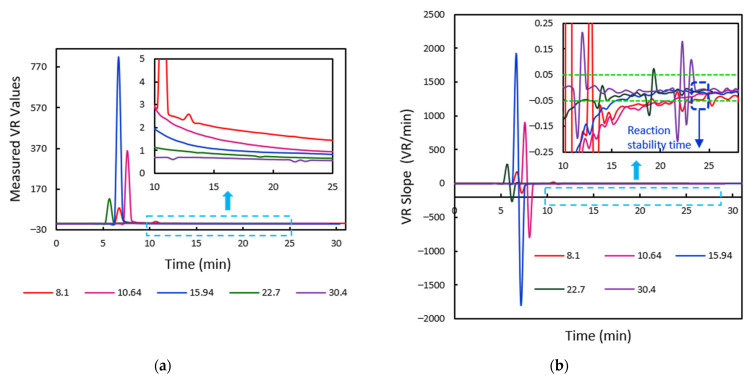
(**a**) The volume ratio values measured for 5 Vitamin D test cartridges of different concentrations. The inset image indicates the zoomed-in VR graph from time t = 10 min to t = 25 min. (**b**) The corresponding trend of change in VR slopes with time, the zoomed graph from time t = 10 min to t = 28 min, is depicted.

A similar analysis was done for Vitamin D using serum samples to demonstrate the evaluation for the reaction time algorithm. Image samples were acquired for 30 min. The trend of the VR values and the corresponding slope plots obtained with respect to time is indicated in Figure 18. It can be noted that the VR slopes are zero for the initial few frames. This duration includes the time for the sample front to reach the end of the membrane as well as the time before the test line forms, thus making the volume ratios zero. Once the test and control regions are detected, the reaction progress is observed, and the slopes can be seen to be changing rapidly. The VR rate for 95% of the samples falls within the ±0.05 band at around 24 min. This is the reaction stability time for Vitamin D samples of the current set of cartridges used. Quantitative measurements for clinical diagnostics must be made at this time.

#### 3.2.3. Calibration of Sample Cartridges and Performance Analysis

**Figure 19:**
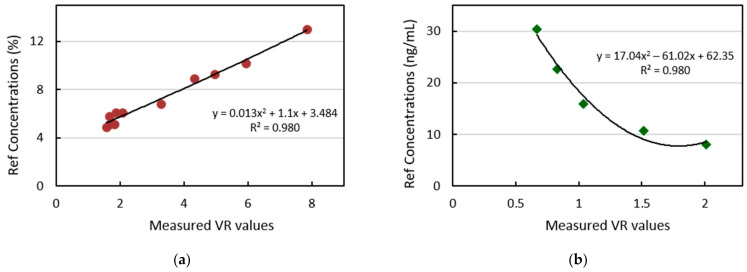
The calibration curve obtained for (**a**) HbA1C test samples with the VR values measured at time t = 12 min (**b**) Vitamin D test samples with the VR values measured at time t = 24 min.

Once the stability time is determined, calibration of the test concentrations is performed by the kit manufacturers. The VR values measured for the samples under test at the reaction stability time are used to establish the calibration of sample cartridges. Figure 19a shows the calibration curve attained for the HbA1C test samples with varying concentrations in the range of 4–14%. As the stability time was determined to be 12 min, the volume ratio values measured at this time instant are used for calibration. Similarly, the calibration curve is obtained for Vitamin D test samples with the VR values measured at time t = 24 min. Five different Vitamin D samples of 8.1–30 ng/mL concentrations are used for setting the calibration curve for demonstration purposes, as indicated in Figure 19b. In practice, the kit manufacturer could use an increased number of samples to improve the calibration accuracy.

The performed calibration is verified by measuring the concentrations of reference HbA1C and Vitamin D samples. The measured VR values are fed to the calibration equations, and corresponding concentrations are determined and compared with the expected values. The experimental study is performed with six HbA1C and three Vitamin D samples. The results obtained for error analysis are indicated in Table 1 and Table 2. The measured concentrations fall within a ±8% error band.

#### 3.2.4. Validation for Abnormality Detection in Test Cartridges

As mentioned in Section 2.2.5, the developed algorithm can detect any irregularities in the test strip. During the development phase, this helps the manufacturer discard the improper test strips from being used for the calibration. To evaluate this, the algorithm is fed with image frames captured for different abnormalities and the proper images. Finally, the algorithm gives output on the condition of the test strip. The metrics sensitivity, specificity, and accuracy of the algorithm are determined as indicated.

(10) $$\%\mathrm{Sensitivity}=\frac{\mathrm{TP}}{\mathrm{TP}+\mathrm{FN}}\;\times \;100$$

(11) $$\%\mathrm{Specificity}=\frac{\mathrm{TN}}{\mathrm{TN}+\mathrm{FP}}\;\times \;100$$

(12) $$\%\mathrm{Accuracy}=\frac{\mathrm{TP}+\mathrm{TN}}{\mathrm{Total}}\;\times \;100$$

where True Positive (TP) is the number of test strips without any irregularities classified as ‘proper’, False positive (FP) is the number of abnormal strips identified as ‘proper’ strips. Similarly, True Negative (TN) is the number of abnormal strips correctly identified as ‘improper’, and False Negative (FN) is the number of proper strips wrongly identified as ‘improper’. These metrics are evaluated separately for flow-related abnormalities and NC membrane irregularities. For detecting abnormalities in sample flow, a total of 16 image samples were fed, with 10 proper cartridges and 6 samples with the skewed flow as mentioned in Section 2.2.5. The corresponding results obtained are indicated in Table 3.

A similar analysis for detecting irreguarities in the NC membrane is done by feeding 120 image frames of proper test strips and 10 frames with bright/dark regions in the NC membrane. The corresponding results are as discussed in Table 4. We can observe that the current algorithm can detect abnormalities with reliable orders of accuracy.

## 4. Conclusions

The developed algorithm, along with the mentioned hardware setup, forms a portable, simple-to-use evaluation tool, facilitating the research involved in the design and manufacturing of fluorescence-labelled quantitative lateral flow immunoassay test cartridges. The presented algorithm practically analyzes the sample flow rates through the test membrane. The algorithm automatically locates the test and control reaction regions within the captured image frames. The tool efficiently detects the presence of any abnormalities, which could be flow-related or irregularities in the manufactured test membranes. It also helps to determine the reaction time required for the sample reaction, providing a visualization of the reaction progress at the test and control lines. Further experimental evaluation of the algorithm is to be carried out with different sample analytes. During the production run as well, this tool can be used to verify the quality of cartridges. The designed tool can also be adopted in an LFIA fluorescence reader for diagnostic measurements, which determines the quantitative estimate of the samples and detects the presence of any abnormalities in the LFIA test cartridges. This brings down the level of human intervention and thereby provides a reliable, practical solution for increased automation in the field of medical diagnostics.

## Figures and Tables

**Table 1:** Measured HbA1C concentrations (%) from the calibration equation.

Expected HbA1C Concentrations (%)	Measured VR Values	Measured HbA1C Concentrations (%)	Relative Error (%)
4.5	1.1099	4.7210	4.91
4.7	1.1316	4.7454	0.97
5	1.2559	4.8860	−2.28
5.7	1.5899	5.2657	−7.62
6.2	2.7497	6.6069	6.56
9	4.3867	8.5598	−4.89

**Table 2:** Measured vitamin D concentrations (ng/mL) from the calibration equation.

Expected Vitamin D Concentrations (%)	Measured VR Values	Measured Vitamin D Concentrations (%)	Relative Error (%)
15.71	1.0655	16.6800	6.16
17.13	1.0501	17.0624	−0.39
29.9	0.6209	31.0314	3.78

**Table 3:** Flow abnormality detection.

No. of Samples for Flow Abnormality Detection		Expected Outcome	
		Proper	Improper
Test Outcome	Proper	10 (TP)	0 (FP)
	Improper	0 (FN)	6 (TN)
% Flow Sensitivity		100	
% Flow Specificity		100	
% Flow Accuracy		100	

**Table 4:** Detection of abnormalities in NC Membrane.

No. of Samples for Detection of Irregularities in NC Membrane		Expected Outcome	
		Proper	Improper
Test Outcome	Proper	116 (TP)	1 (FP)
	Improper	4 (FN)	9 (TN)
% Irregularity Detection Sensitivity		96	
% Irregularity Detection Specificity		90	
% Irregularity Detection Accuracy		96	

## Data Availability

Not applicable.
